# Chloroplast genome of critically endangered *Cotoneaster wilsonii* (Rosaceae) endemic to Ulleung Island, Korea

**DOI:** 10.1080/23802359.2019.1687361

**Published:** 2019-11-08

**Authors:** Ji Young Yang, Jae-Hong Pak, Seung-Chul Kim

**Affiliations:** a Research Institute for Dok-do and Ulleung-do Island, Department of Biology, School of Life Sciences, Kyungpook National University, Daegu, Republic of Korea;; b Department of Biological Sciences, Sungkyunkwan University, Suwon, Republic of Korea

**Keywords:** Chloroplast genome, endangered species, *Cotoneaster wilsonii*, Ulleung Island, Rosaceae

## Abstract

*Cotoneaster wilsonii* Nakai is an endangered species endemic to Ulleung Island, Korea. Here we reported the first complete chloroplast gnome sequences of *C. wilsonii*, which is 159,999 bp in total length with the large single copy (LSC) region of 87,868 bp, the small single copy (SSC) region of 19,335 bp, and two inverted repeat (IR) regions of 26,399 bp. The plastome contains 131 genes, including 84 protein-coding, eight ribosomal RNA, and 37 transfer RNA genes. The overall GC content is 42.6% and those in the LSC, SSC, and IR regions are 34.2, 30.3, and 42.6%, respectively. Phylogenetic analysis of 21 representative plastomes within the family Rosaceae suggests strongly the monophyly of *Cotoneaster* and *C. wilsonii* being sister to the clade of *Cotoneaster franchetii* and *Cotoneaster horizontalis*.

The genus *Cotoneaster* Medik. (Rosaceae) contains numerous ornamentally important shrubs and small trees, and consists of ca. 90 species widely distributed in the northern hemisphere, with the center of diversity in the Himalayas and western China (Yü 1974; Willis 1985; Fryer and Hylmö 2009). The majority of species (ca. 90%) are apomictic and tetraploid, and relationships within *Cotoneaster* are poorly known due in part to many species complex groups and considerable morphological variation (Kroon 1975; Bartish et al. 2001; Talent and Dickinson 2007; Fryer and Hylmö 2009; Dickoré and Kasperek 2010). The earliest classification system based primarily on petal characters (Koehne 1893) resulted in recognization of two subgenera, and several other classification systems have recently been proposed (Yü 1963; Flinck and Hylmö 1966; Phipps et al. 1990; Fryer and Hylmö 2009). Despite several attempts to determine phylogenetic relationships among species, we know little about interspecific relationships within *Cotoneaster* (Bartish et al. 2001; Lo and Donoghue 2012; Li et al. 2014). In the Korean Peninsula, two species of *Cotoneaster*, *Cotoneaster wilsonii* and *Cotoneaster integerrimus* Medik., are known to occur. While *C. integerrimus* is known to occur only in North Korea, *C. wilsonii* is endemic to Ulleung Island, which is young oceanic and volcanic island in East Sea. About 100 individuals of *C. wilsonii* are found in three subpopulations of sunny cliffs at 110–130 m above sea level, and designated as critically endangered species (CR B2ab(ii)) (National Institute of Biological Resources 2014). Although Chang and Jeon (2003) questioned a distinct species status of *C. wilsonii* based on flavonoids and morphology, overall species relationships within *Cotoneaster multiflorus* complex remain to be determined.

In this study, we sequenced the complete plastome of *C. wilsonii*, and assessed its phylogenetic position within Rosaceae. The collecting permit was obtained via the Division of Forest Biodiversity of Korea National Arboretum. Total genomic DNA (voucher specimen: SKU-Yang1804025; N37°29′9′′ E130°54′28′′) was isolated from fresh leaves using the DNeasy Plant Mini Kit (Qiagen, Carlsbad, CA). Genome sequencing was conducted using the Illumina HiSeq 4000 (Illumina Inc., San Diego, CA). A total of 31,268,870 pair-end reads were obtained and assembled de novo with Velvet v. 1.2.10 using multiple k-mer (Zerbino and Birney 2008). The tRNAs were confirmed using tRNAsacn-SE (Lowe and Eddy 1997). The complete plastome sequence of *C. wilsonii* (Genbank accession number: MN516695) was 159,999 bp, with large single copy (LSC; 87,868 bp), small single copy (SSC; 19,335 bp), and two inverted repeats (IR_a_ and IR_b_; 26,399 bp each). The overall GC content was 42.6% (LSC, 34.3%; SSC, 30.3%; IRs, 42.6%) and the plastome contained 131 genes, including 84 protein-coding, 8 rRNA, and 37 tRNA genes. The maximum likelihood (ML) analysis was conducted using IQ-TREE v.1.4.2 (Nguyen et al. 2015) to determine phylogenetic position of *C. wilsonii* based on plastomes of 21 representative species of Rosaceae. The complete plastome sequences were aligned using MAFFT v.7 (Katoh and Standley 2013). The ML tree confirmed that the genus *Cotoneaster* is monophyletic and showed that *C. wilsonii* is sister to *Cotoneaster franchetii* and *Cotoneaster horizontalis* clade (Figure 1).

**Figure 1.**
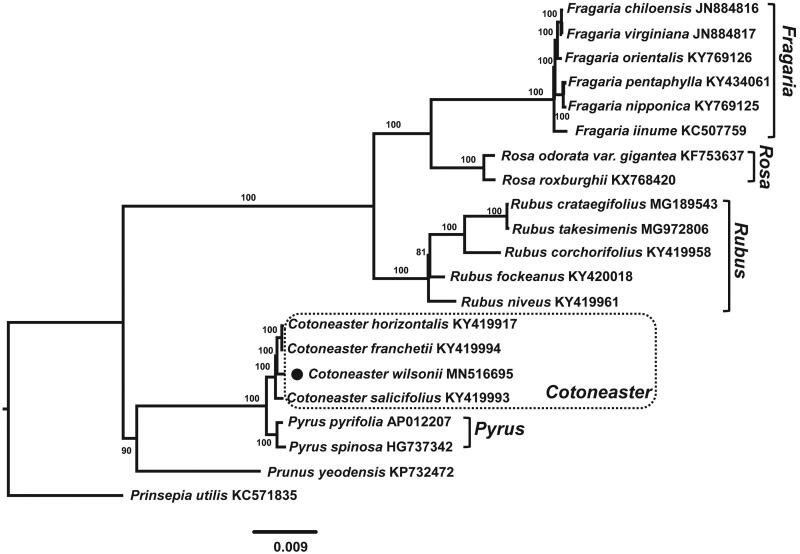
The maximum-likelihood (ML) tree based on the 21 representative chloroplast genomes of Rosaceae. The bootstrap value based on 1000 replicates is shown on each node.
